# Low Molecular Weight and Polymeric Modifiers as Toughening Agents in Poly(3-Hydroxybutyrate) Films

**DOI:** 10.3390/polym12112446

**Published:** 2020-10-22

**Authors:** Adriana Nicoleta Frone, Cristian Andi Nicolae, Mihaela Carmen Eremia, Vlad Tofan, Marius Ghiurea, Ioana Chiulan, Elena Radu, Celina Maria Damian, Denis Mihaela Panaitescu

**Affiliations:** 1 Polymer Department, National Institute for R&D in Chemistry and Petrochemistry ICECHIM, 202 Splaiul Independentei, 060021 Bucharest, Romania; ca_nicolae@yahoo.com (C.A.N.); ghiurea@gmail.com (M.G.); ioana.chiulan@icechim.ro (I.C.); nina.radu58@yahoo.ro (E.R.); 2 National Institute for Chemical Pharmaceutical Research and Development ICCF, 112 Calea Vitan, 031299 Bucharest, Romania; mihaelaceremia@yahoo.com; 3 Cantacuzino National Institute of R&D for Microbiology and Immunology, 103 Splaiul Independentei, 050096 Bucharest, Romania; tofan.vlad@gmail.com; 4 Advanced Polymer Materials Group, Faculty of Applied Chemistry and Materials Science, University Politehnica of Bucharest, 1-7 Gheorghe Polizu, 011061 Bucharest, Romania; celina.damian@yahoo.com

**Keywords:** poly(3-hydroxyoctanoate), polyhydroxybutyrate, bio-based modifiers, toughening, biocompatibility, thermal properties

## Abstract

The inherent brittleness of poly(3-hydroxybutyrate) (PHB) prevents its use as a substitute of petroleum-based polymers. Low molecular weight plasticizers, such as tributyl 2-acetyl citrate (TAC), cannot properly solve this issue. Herein, PHB films were obtained using a biosynthesized poly(3-hydroxyoctanoate) (PHO) and a commercially available TAC as toughening agents. The use of TAC strongly decreased the PHB thermal stability up to 200 °C due to the loss of low boiling point plasticizer, while minor weight loss was noticed at this temperature for the PHB-PHO blend. Both agents shifted the glass transition temperature of PHB to a lower temperature, the effect being more pronounced for TAC. The elongation at break of PHB increased by 700% after PHO addition and by only 185% in the case of TAC; this demonstrates an important toughening effect of the polymeric modifier. Migration of TAC to the upper surface of the films and no sign of migration in the case of PHO were highlighted by X-ray photoelectron spectroscopy (XPS) and atomic force microscopy (AFM) results. In vitro biocompatibility tests showed that all the PHB films are non-toxic towards L929 cells and have no proinflammatory immune response. The use of PHO as a toughening agent in PHB represents an attractive solution to its brittleness in the case of packaging and biomedical applications while conserving its biodegradability and biocompatibility.

## 1. Introduction

A huge percentage close to 90% of the globally produced plastics uses virgin fossil fuel feedstocks [1]. It is estimated that plastics’ share will grow to around 20% of the global oil consumption by 2050 if this trend continues [1]. In this context, the plastics market has turned to polymers derived from renewable resources as eco-friendly alternatives to petroleum-based polymers. Poly(3-hydroxybutyrate) (PHB) belongs to the large family of polyhydroxyalkanoates (PHAs), which are fully biodegradable biopolyesters of hydroxycarbonic acids produced by either chemical or bacterial synthesis from biorenewable and biowaste resources [2,3]. Due to its biodegradability, biocompatibility, and competitive physical properties, PHB is the most well studied PHA for biomedical and food packaging materials [4,5,6]. However, its high production cost and advanced brittleness narrow its application as a substitute for common synthetic polymers [4,5,6,7]. Plasticizers may increase PHB flexibility and toughness, thus solving the problem related to its high brittleness [4,7,8,9,10,11,12,13,14,15]. Tributyl 2-acetylcitrate (TAC) is obtained from naturally occurring citric acid. It may be used as a plasticizer for applications starting from food packaging to biomedical applications as it is environmentally friendly and, in small amounts, does not raise safety concerns for humans [14,16,17,18]. Nevertheless, a major issue when using TAC or other citrate esters as plasticizers is related to their relatively low boiling point, which results in considerable weight loss at temperatures from 160 to 200 °C, therefore within the processing temperature range of PHB [19,20]. Moreover, TAC addition could trigger the migration at the surface of the film [13], thus deteriorating the mechanical properties. In particular, Corrȇa et al. [20] explained the decrease in the thermal stability of poly(3-hydroxybutyrate-co-3-hydroxyvalerate)-TAC/organo-modified montmorillonite nanocomposites through the low degradation temperature of neat plasticizer (around 130 °C). Similarly, the addition of only 5 wt% TAC lowered the temperature to 5% weight loss of PHB and also influenced the maximum degradation peak [17]. Maiza et al. [16] observed that triethyl citrate and TAC, used as plasticizers in PLA, migrate out of the matrix, the weight loss being directly proportional to the temperature (100 or 135 °C) and plasticizer concentration. In general, low molecular weight plasticizers are liquids which are not chemically bonded to the polymer matrix and, therefore, at room or elevated temperature, they leach out from the polymer matrix [21]. When plasticized PHB is used for scaffolds and other medical devices or for food packaging, the plasticizer will leach out into the surrounding medium (human body or foods), raising health problems overtime or damaging food quality. In this context, the use of polymeric toughening agents could be a better solution to the inherent brittleness of PHB. Therefore, elastomeric medium chain length PHAs may be a better alternative to TAC and other citrate esters provided they successfully increase the flexibility and other properties of PHB while preventing migration.

Polyhydroxyoctanoate (PHO) belongs to the same PHA family as PHB, but it has a higher side-chain length, much lower crystallinity and melting point, and it exhibits elastomeric properties [22,23]. Therefore, it can provide increased ductility to PHB, reduced migration, and no leakage when processed at high temperatures. However, PHO biopolymer is not compatible with PHB, forming a biphasic system over the whole composition range [24]. Previous studies showed a noticeable increase in the elongation at break only from a high amount of PHO in the blends [22,24]. Thus, a doubling of the elongation at break was observed at 20 [24] or 15 wt% [24] PHO in PHB/PHO blends, depending on PHO type and preparation conditions, together with a strong decrease in tensile strength and modulus [22,24]. In biocomposites with a low amount of PHO (5 wt%), the supplementary addition of bacterial cellulose restored the Young’s modulus [25]. Therefore, the domain of low PHO concentrations is more interesting from both a scientific and economical point of view and this was thoroughly investigated in the case of PHB/PHO blends obtained by solution casting.

In this work, we synthesized a PHO homopolymer (95 mol% 3-hydroxyoctanoate units) and we comparatively studied the toughening effect of PHO and low molecular weight TAC in neat PHB. PHB films with different concentrations of the two bio-based modifiers were characterized to obtain information on the surface morphology, crystalline structure, biocompatibility, and thermal and mechanical properties, useful for the intended application in packaging and biomedicine.

## 2. Materials and Methods

### 2.1. Materials

PHB powder with density of 1.24 g/cm^3^ was acquired from Biomer(Schwalbach am Taunus, Germany) and was used as received. Tributyl 2-acetylcitrate ≥98% was purchased from Sigma-Aldrich. Chloroform used for polymer dissolution was supplied by a local company (Chimopar SRL, Bucharest, Romania).

### 2.2. Biosynthesis of PHO

*Pseudomonas fluorescens* ICCF 392 strain from ICCF Culture Collection of Microorganisms was used for the biosynthesis of PHO. Thestrain *Pseudomonas fluorescens* was isolated from 1 g of rotten beech wood powder, homogenized in 20 mL sterile broth. The pre-inoculum culture (10%, *w*/*v*) was maintained on a specific medium consisting of (*w*/*v*) yeast extract 1.0%, peptone 1.0%, glycerol 5.0%, and agar 2%. The composition of culture medium for inoculum (IPS medium) was glucose 1 g/100 mL, corn extract 1.5 g/100 mL, KH_2_PO_4_ 1 g/100 mL, NaCl 1 g/100 mL, and MgSO_4_ 0.05 g/100 mL. Inoculum culture was developed in Erlenmeyer flasks of 500 mL under stirring at around 30 °C, for 24 h. The fermentation medium was inoculated with 10% (*w*/*v*) of inoculum. Besides the carbon source (sodium octanoate 2.0 g/L), the culture medium used in the fermentation process contained NaNH_4_HPO_4_·4H_2_O 3.5 g/L, K_2_HPO_4_ 7.5 g/L, KH_2_PO_4_ 3.7 g/L, citric acid 20 g/L, 0.1mL/100mL of trace element solution 1, and 0.1mL/100mL of solution 2. Trace element solution 1 consisted of (per liter 1M HCl) MgSO_4_·7H_2_O, 120.0 g. Trace element solution 2 consisted of (per liter 1M HCl) FeSO_4_·7H_2_O, 2.78 g, CaCl_2_·2H_2_O, 1.47 g, MnCl_2_·4H_2_O, 1.98 g, CoSO_4_·7H_2_O, 2.81 g, CuCl_2_·2H_2_O, 0.17 g, and ZnSO_4_·7H_2_O, 0.29 g. Bioprocesses were carried out in 500 mL flasks containing 100 mL of culture broth, which were maintained on a rotary shaker at 220 min^−1^, 29 ± 1 °C for 48 h. The cultivation was performed by nutrient addition (sodium octanoate stock solution, 83.33 g/L) with the sequences of 3 mL each at 0, 24, and 30h, respectively. A total biomass of 1.975 g dry cells/L of fermentation medium was obtained and the yield of PHO was 21.10 g/100 g dry biomass.

### 2.3. Preparation of PHB/PHO and PHB/TAC

The PHB/PHO films were prepared using a solvent-casting method. Dried PHO membrane was dissolved in chloroform and stirred at room temperature until complete dissolution. PHB powder was subsequently added, so as to obtain different PHB:PHO weight ratios: 100:0 (PHB), 95:5 (PHB/5PHO), 90:10 (PHB/10PHO), 85:15 (PHB/15PHO), and 80:20 (PHB/20PHO). The concentration of the total amount of polymers in the chloroform was kept constant at 4 wt%/v. The resulting mixture was stirred at room temperature for 10 min until the polymers were well dispersed and then heated at 50 °C for 1 h to ensure complete dissolution of both components. PHB/PHO films with a thickness of around 10 µm were cast from the resulting solutions onto glass slides. They were initially dried at room temperature for several hours and then in a vacuum oven at 45 °C for 48 h to remove any residual solvent. PHB/TAC films with the same weight ratios as for PHB/PHO films were prepared following the same procedure and denoted as PHB/5TAC, PHB/10TAC, PHB/15TAC, and PHB/20TAC, while PHB film served as a reference.

### 2.4. Characterization

#### 2.4.1. Gas Chromatography–Mass Spectrometry (GC-MS) Analysis of PHO

Triple Quad GC/MS system (Agilent Technology) was used for both qualitative and quantitative analysis of the PHO monomers. First, 0.5 μL of PHO polyester sample was injected using the split mode and the injector temperature was set to 250 °C. The analysis was performed using a HP-FFAP DB-WAX column (30 m × 0.25 mm, with a 0.25 μm film thickness) with a mobile phase of helium at a constant flow rate of 1 mL min^−1^. The GC oven temperature was held for 1 min at 90 °C, then raised by 7 °C/min up to 240 °C and held for 12 min. MS transfer temperature was set to 280 °C, MS ion source to 230 °C. The electron ionization (EI) source of the instruments was operated at 70 eV in the scan range (*m*/*z*) = 40–400. The fragmentation pattern of the obtained mass spectra was analyzed by NIST 98 Mass Spectral Database, Gaithersburg, MD, USA.

#### 2.4.2. Thermogravimetric Analysis (TGA)

Thermogravimetric analysis (TGA) of the blends was carried out on a TA Q5000 analyzer (TA Instruments Inc., New Castle, DE, USA), from 25 to 700 °C, with 10 °C/min, using nitrogen as a purge gas (40 mL/min).

#### 2.4.3. Differential Scanning Calorimetry (DSC)

Thermal parameters of the blends, including glass transition temperature (*T_g_*), melting temperature (*T_m_*), crystallization temperature (*T_c_*), their specific enthalpies and crystalline ratio (*X_c_*), were determined with a DSC Q2000 calorimeter (TA Instruments Inc., New Castle, DE, USA) under helium flow (25 mL/min). A heat–cool modulation program from −65 to 190 °C, at an average heating/cooling rate of 10 ± 0.80 °C/min with a period of 30 s, was used. The crystallinity degree of PHB in the films was estimated from the melting enthalpy values (Δ*H_m_*) of the samples and the melting enthalpy of 100% crystalline PHB ($\Delta H_{m}^{0}$, 146 J/g) [25] using Equation (1).

(1) $$X_{c}\left[\%\right]\;=\;\frac{\Delta H_{m}}{\Delta H_{m}^{0}\times w_{i}}\;\times \;100$$

where *w_i_* is the weight fraction of PHB in the blends.

#### 2.4.4. X-ray Diffraction (XRD)

The crystalline phase of neat PHB and PHB blends was analyzed using an X-ray diffractometer (Rigaku Corporation, Japan) with a Cu Kα (λ = 0.1541 nm) source. Scanning was performed at 45 kV and 200 mA at the 2θ scanning angle, between 5° and 40°, with a scanning step of 0.04 min^−1^. The degree of crystallinity (CI) of the samples was calculated using Equation (2), where *A_C_* is the sum of the areas under the crystalline peaks, and *A_a_* is the area of the amorphous halo.

(2) $$\mathrm{CI}\;\left(\%\right)\;=\;\frac{A_{C}}{A_{c\;}+A_{a}}\;\times \;100$$

The inter-planar distances (d) were calculated using Bragg’s law [26] and the crystallite sizes (D) at the main peaks using the Scherrer equation [27]. Fityk software was used for XRD data processing and nonlinear curve fitting.

#### 2.4.5. Tensile Tests

Tensile tests were conducted according to ISO 527, Part 3, which is applicable to films, at room temperature using an Instron 3382 universal testing machine with a load cell of 1 kN. For each sample, at least 5 specimens of type 5A (10 µm thickness) were tested with a crosshead speed of 2 mm min^−1^. The average values and the standard deviations for Young’s modulus, tensile strength, and elongation at break were calculated using the Bluehill 2 Software.

#### 2.4.6. Atomic Force Microscopy (AFM)

The surface morphology and the root mean square roughness of solvent-cast PHB films surfaces were determined using a MultiMode 8 atomic force microscope (Bruker, Santa Barbara, CA, USA). The characterization of each sample was performed in Peak Force (PF) Quantitative Nanomechanical Mapping (QNM) mode, in air, using silicon nitride tips at a scan rate of 1 Hz and a scan angle of 90°. The image processing and data analysis were conducted with NanoScope software version 1.20.

#### 2.4.7. X-ray Photoelectron Spectroscopy (XPS)

The chemical composition at the surface of neat PHB and plasticized films was analyzed using a fully integrated K-Alpha system (Thermo Scientific, Waltham, MA, USA) equipped with a monochromated AlK_α_ source (1486.6 eV). Both survey (0–1200 eV) and high-resolution spectra were recorded for PHB blends. Charging effects were compensated by a flood gun and binding energies were calibrated by placing the C1s peak at 284.8 eV as internal standard. The pass energy for the survey spectra was set to 200 eV, and for the high-resolution spectra, it was 20 eV.

#### 2.4.8. Contact Angle Measurements (CA)

CA measurements were carried out using a CAM 200 instrument (Biolin Scientific, Gothenburg, Sweden) equipped with a high-resolution camera (Basler A602f) and an auto-dispenser. CA was measured in air, at room temperature and ambient humidity, 2 s after the drop contacted the surface of the films. Drops of 6 μL deionized water were dispensed on each sample, and the value of the reported CA was the average of seven measurements. The images of the droplets were acquired with the high-resolution camera using CAM software.

#### 2.4.9. Biocompatibility Test

Biocompatibility of PHB blends was tested using fluorescence microscopy as described elsewhere [25]. Film samples were sterilized by incubation in 70% ethanol solution overnight and then washed with sterile phosphate buffer saline (PBS) to discard residual ethanol. Afterwards, 0.32 cm^2^ sterilized discs were carved out of each film and deposited in an ultra-low attachment flat-bottom 96-well plate (Sigma-Aldrich) to prevent cell adhesion to surfaces other than those tested. L929 murine fibroblast cells were seeded at a concentration of 1 × 10^4^ cells/well on top of each sample and allowed to adhere for two different time intervals (1 and 9 days). After each incubation period, samples were gently washed to discard non-adherent cells. Further, samples were fixed with 4% paraformaldehyde (Sigma-Aldrich) solution, followed by staining with staining solution: 0.05% Triton X-100, SYBR-Green I, Texas-Red-X Phalloidin (Molecular Probes, Thermo Fisher Scientific). Images were recorded with an Eclipse TE2000 inverted fluorescence microscope (Nikon, Austria) and processed with Huygens software (SVI, Hilversum, The Netherlands).

#### 2.4.10. Evaluation of Pro-Inflammatory Effect

Nearly equal amounts of each film were sterilized by overnight incubation in 70% ethanol solution followed by washing steps with PBS to completely remove ethanol. Samples were incubated at 37 °C in PBS for 20 days to allow elution of their constituents. A sample of PBS alone, incubated in a similar fashion, served as a control. Following incubation, elution samples were collected and tested for endotoxin activity with LAL QCL-1000 kit (Lonza, Verviers Belgium). Further, differentiated macrophage-like cells were incubated in the presence of collected elution samples (diluted 1/10 in complete culture medium) for 24 h. Cells incubated with lipopolysaccharide (LPS 100 ng/mL) or without stimuli were used as positive and negative controls. Supernatants were collected and stored at −80 °C until use. The influence of elution samples on differentiated macrophages was evaluated by measuring tumor necrosis factor-α (TNF-α) concentrations in culture supernatants using ELISA (DuoSet kits from R&D Systems Inc., Minneapolis, MN, USA).

## 3. Results and Discussion

### 3.1. PHO Characterization

**Figure 1:**
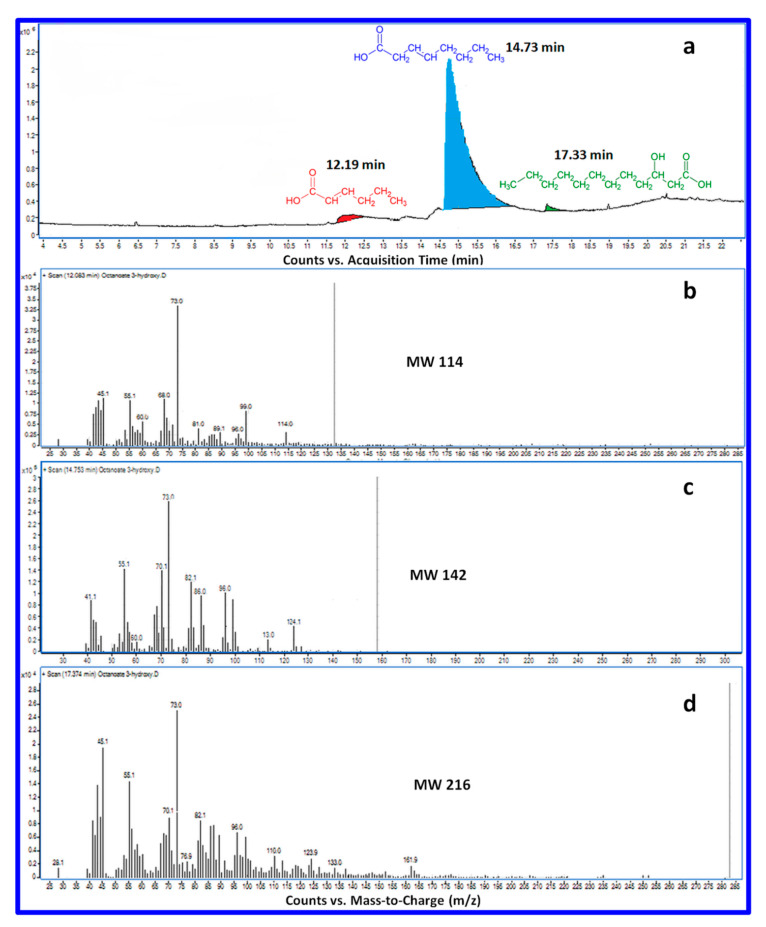
GC-MS chromatogram of PHO synthesized from *Pseudomonas fluorescens* showing monomeric composition of PHO (**a**) and mass spectra of trans-2-hexenoic acid (**b**), 3-octenoic acid (**c**), and 3-hydroxy-dodecanoic acid (**d**).

PHO was characterized in terms of the monomer structure and composition by GC-MS analysis through its conversion to volatile carboxylic acids (Figure 1). The gas chromatogram showed a major peak with the retention time of 12.19 min and two minor peaks at 14.73 and 17.33 min (Figure 1a). The major peak corresponds to 3-octenoic acid, the first minor peak corresponds to trans-2-hexenoic acid, while the second minor peak was identified as 3-hydroxy-dodecanoic acid by comparing molecules in the GC database. Therefore, the biosynthesized PHO was mostly composed of 3-hydroxyoctanoate (3HO—C8), which represents 95.02 mol% of the total monomer content and very small amounts of 3-hydroxyhexanoate (3HHx—C6, 3.32 mol%) and 3-hydroxydodecanoate (3HDD—C12, 1.66 mol%) (Figure 1b–d). All products were detected in the form of single-type monomers only.

In addition, DSC analysis of PHO displayed a *T_g_* at −35 °C and a double melting event with a major melting peak at around 43 °C and a shoulder at 51 °C. The total enthalpy of fusion was 16.22 J/g, suggesting a low degree of crystallinity of only 11% if the melting fusion of 100% crystalline PHB was used.

### 3.2. Thermal Stability of PHB Blends

**Figure 2:**
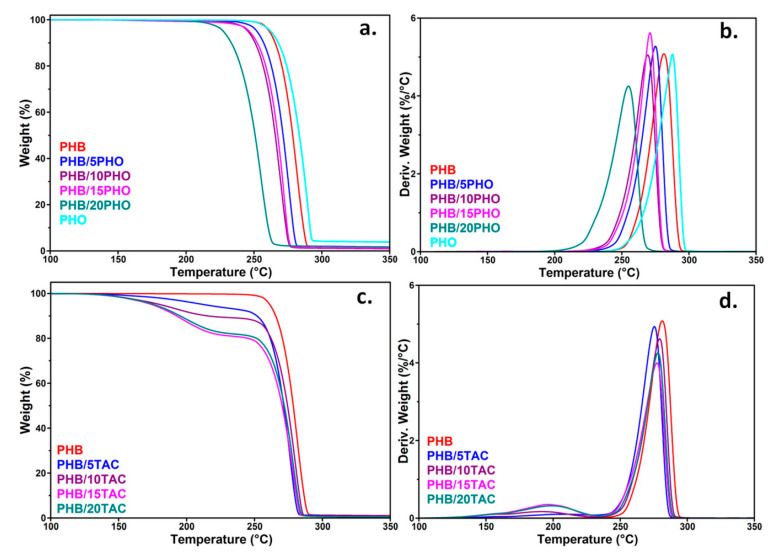
TGA and DTG curves for neat PHB, PHO (**a**,**b**) and TAC (**c**,**d**) plasticized PHB films.

The thermal degradation of neat PHB and PHB blends was investigated by TGA and the thermograms are plotted in Figure 2a–d, while the main parameters resulting from TGA and derivative curves (DTG) are summarized in Table 1. The thermal degradation of neat PHB and PHB/PHO films took place in a single stage, whereas that of PHB/TAC films showed two separated weight loss steps (Figure 2a–d). The temperature at which PHB/PHO films lost 5% of their mass (*T_5%_*) exhibited a downshift of 10–15 °C for up to 15 wt% PHO and of 33 °C for PHB/20PHO. A similar behavior was observed in the case of the temperature of the maximum decomposition rate (*T_d_*), which decreased by less than 10 °C for up to 15 wt% PHO and by around 26 °C at maximum PHO concentration (Table 1). This major degradation step may be ascribed to the random chain scission of PHB ester bonds by intramolecular cis-elimination, where degradation products like crotonic acid, linear oligomers with a crotonate end-group, and dimers and trimers of crotonic acid were formed [17,28].

The thermal stability of PHO is similar to that of PHB or better (Figure 2a). Therefore, the degradation of PHB/PHO may be determined by the presence of impurities, knowing that sodium and other metal salts are common elements in PHO biosynthesis and the thermal stability of PHB is very sensitive to metal traces [29,30]. Thus, it was reported that the residual metal compounds, derived from the fermentation process, catalyzed the depolymerization of PHB, resulting in a depression of the thermal stability [29,30]. Moreover, the difference observed between the thermal stability of PHB with up to 15 wt% PHO and that with 20 wt% PHO could indicate increased incompatibility and phase segregation at the highest concentration of PHO.

As for PHB/TAC films, the first degradation step was observed at around 220 °C and was attributed to the vaporization of most of the TAC plasticizer, while the second step, from 230 to 300°C, corresponds to PHB degradation [14]. Indeed, the weight loss at 220 °C (*WL*_200_), around 5, 7, 17, and 17%, was close to the corresponding proportion of TAC in plasticized PHB (5, 10, 15, and 20%) (Table 1). The differences may come from some vaporization of TAC during the melt processing step. Moreover, much lower *T*_5%_ values compared to those of PHB/PHO films were noticed, the differences between the corresponding compositions ranging from 39 to 72 °C (Figure 2c vs. Figure 2a). Compared to PHB, the decrease in *T*_5%_ was higher, with 85 °C for the maximum TAC concentration (Table 1). The differences come from the low boiling point of TAC plasticizer [16,19,20], the decrease in the thermal stability of PHAs due to the vaporization of low molecular weight plasticizers being previously reported [9,10]. In addition, the *T_d_* values, corresponding to the thermal degradation of PHB, remained close to each other and to that of neat PHB (Table 1, Figure 2d) due to the evaporation of most of the TAC at up to 220 °C.

### 3.3. DSC Analysis of Plasticized PHB

**Figure 3:**
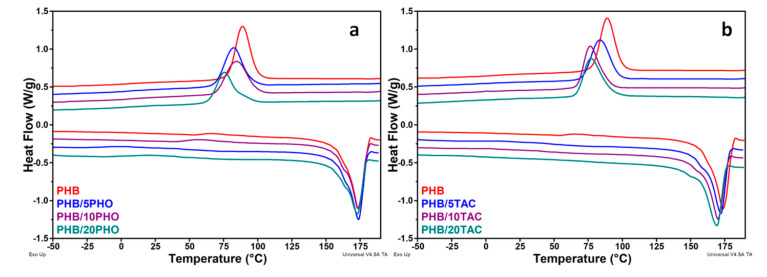
DSC first heating and cooling curves for neat PHB and PHB containing PHO (**a**) and TAC (**b**).

The influence of PHO and TAC on the melting and crystallization behaviors of PHB films was investigated by DSC. The values for the main thermal events, namely the glass transition temperature (*T_g_*), melting (*T_m_*) and crystallization (*T_c_*) temperatures, their enthalpies, and the crystallinity degree (*X_c_*), were reported in Table 2. Neat and plasticized PHB films displayed a well-defined melting peak with a shoulder at a lower temperature (Figure 3a,b). Double or multiple melting behaviors have been previously reported for PHB and were related either to the process of partial melting, recrystallization, and remelting or to the melting of crystals with different crystalline structures, perfection, or thickness [9,31]. Thus, the shoulder at low temperature (*T_m_*_1_) could be associated with the fusion of the crystals with low perfection and thinner lamella and the main fusion peak at a higher temperature (*T_m_*_2_) to the fusion of more perfect (recrystallized) crystals [32,33]. Another assumption related to the double melting peak is the presence of β crystals that melt at a lower temperature than the common α form of PHB [34].

PHB/PHO films showed similar thermal behavior with that of neat PHB (Table 2), suggesting a low influence of PHO on the mobility of PHB chains in the melting region due to the incompatibility of PHB and PHO in this temperature range. On the contrary, TAC addition induced a systematic and clear depression of *T_m_*_1_ values, with 5–13 °C, along with its increasing content in PHB. A less important shift, between 2 and 5 °C, was noticed for the main melting peak (Table 2). These observations indicate that the presence of TAC favors the segmental motion of the PHB chains, especially of those with lower molecular weight. It must be noted that the area of the main peak (*T_m_*_2_) decreased notably in the case of PHB/PHO films and to a lesser extent for PHB/TAC ones (Table 2). This can be an indication of smaller population of more perfect and larger crystals in PHB films containing PHO as against PHB/TAC films.

The *T_g_* values were determined from the first cooling scan (Figure S1, Supplementary Materials). A decrease in the glass transition temperature of PHB was noticed in all the blends and it depended on both the concentration and the type of modifier. *T_g_* was shifted from 7.1 °C for neat PHB to 4.0 °C for PHB/20PHO and to −1.5 °C for PHB/20TAC (Table 2). The smaller decrease in *T_g_* in the case of PHB/PHO compared to PHB/TAC may be due to the lack of miscibility and the segregation of PHB and PHO domains following their melting during the previous heating cycle. A similar decreasing trend in *T_g_* values was reported for PHB containing different plasticizers [12,35].

A very broad exothermic event, from around −25 to 75°C, depending on the modifier type and concentration, was noticed for most of the films (Figure 3a,b) and may be related to a cold crystallization event. It was reported that the crystallization behavior of plasticized PHAs composites is a complex process due to several overlapping phenomena [20]. In this context, we assume that the cold crystallization process can overlap with the glass transition, which occurs in the same temperature range, remelting, or branching. However, TAC induced a less pronounced event in PHB films compared to PHO, probably due to the important increase in polymer chain mobility. This is obvious at maximum TAC content, when no such broad exothermic event occurred (Table 2, Figure 3b). No change in crystallinity was noticed in PHB blends compared to neat PHB except for a slight increase in crystallinity for the PHB/20TAC film (Table 2).

During cooling, a downshift of *T_c_* was induced by the presence of both modifiers in PHB (Table 2, Figure 3a,b). For the highest amount of PHO and TAC, the depression of the *T_c_* values was significant, of 13.3 and 11.9 °C, respectively (Table 2). This may be attributed to increased mobility up to a lower temperature. Further, with increasing PHO content in PHB films, *T_c_* peaks became broader, pointing out the presence of a large range of crystallite sizes and possible increased compositional heterogeneity (Figure 3a,b). No major differences were noticed during the second heating cycle as compared to the first one (Figure S2, Supplementary Materials).

Thermal analyses showed different influences of TAC and PHO upon the crystallization of PHB. Thus, due to its low molecular weight and good miscibility with PHB, along with its high volatility, TAC increased the chain mobility of PHB in the amorphous phase through a lubricity effect and leached out from the matrix above 200 °C. Contrarily, PHO was not miscible with PHB, especially in the domain of the melting temperatures when segregation occurred; however, it remained in the composition of PHB films, hindering PHB crystallization.

### 3.4. X-ray Diffraction (XRD)

**Figure 4:**
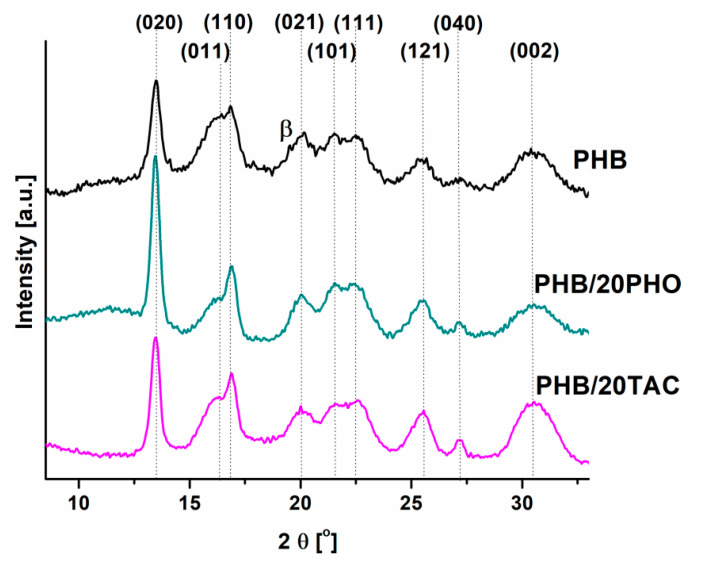
XRD spectra of neat PHB and PHB films containing 20 wt% PHO and TAC.

The XRD spectra of neat PHB and PHB blends with the highest proportion of TAC and PHO are presented in Figure 4.

The diffractograms of neat PHB and PHB blends revealed a similar crystalline profile corresponding to orthorhombic crystal planes [36,37]. Two strong crystalline peaks were detected at 2θ 13.5° and 16.9°, which were assigned to the (020) and (110) planes of the orthorhombic unit cell and several weaker reflections, as shown in Figure 4. All the samples contain also a small amount of orthorhombic β-form crystal with zig-zag conformation, as revealed by the reflection of the (021) plane located at around 2θ = 20.1°. The occurrence of the β crystalline form may contribute to the presence of a shoulder before the melting peak, signaled by DSC analysis. Increased intensity of the peak corresponding to (020) crystal plane was observed in the blends (Figure 4), especially in PHB/20PHO, suggesting the presence of a preferred orientation and increased crystallinity. Indeed, higher CI values were calculated from the XRD data of PHB/PHO (76%) and PHB/TAC (71%) compared to neat PHB (57%). Therefore, PHO contributed to a larger extent to the formation of an ordered crystalline structure in PHB. The differences between the crystallinity values determined by XRD and DSC may come from the difference between the methods, XRD emphasizing the surface crystallinity and DSC that of the bulk material and from the influence of the temperature in DSC heating on the segregation of PHO and PHB domains.

The interplanar distances (*d_hkl_*) and the apparent crystallite sizes perpendicular to the hkl (where h, k, and l are the Miller indices) plane (*D_hkl_*) were estimated using Bragg’s law and the Scherrer equation and the results are summarized in Table 3.

Higher crystal sizes perpendicular to the (020), (110), and (040) planes were observed for the PHB films containing PHO and TAC, similar to other observations [38]. This increase in crystal size may be due to the greater order of the crystal structure after the addition of modifiers.

### 3.5. Mechanical Characterization

**Figure 5:**
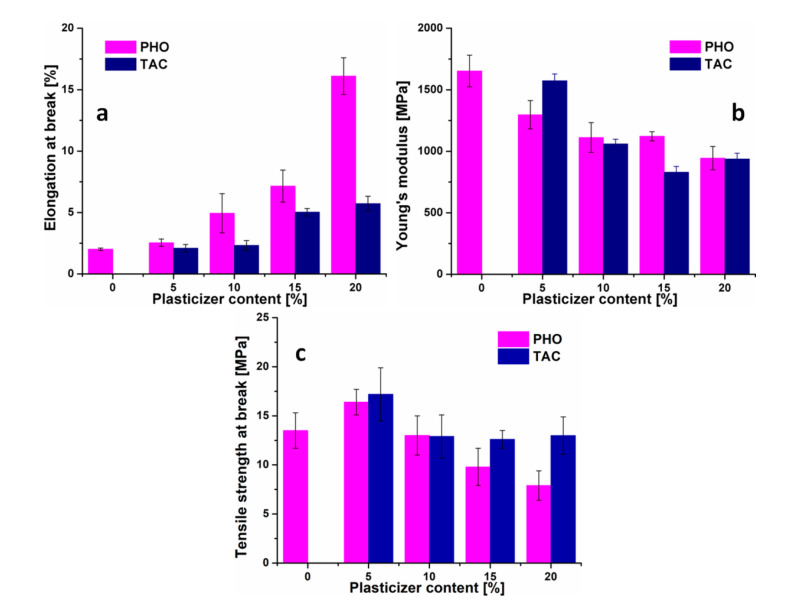
Mechanical properties determined from the tensile tests: elongation at break (**a**), Young’s modulus (**b**), and tensile strength (**c**).

The evolution of the mechanical properties of neat and modified PHB films is shown in Figure 5. Both modifiers increased the flexibility of PHB films in correlation with their amount in the blends but the effect was much more significant in the case of PHO, which emerges as an efficient toughening agent. Thus, at maximum PHO and TAC content, the elongation at break was higher, with 700 and 185%, than that of neat PHB (Figure 5a). This means that PHO induced four times higher ductility. On the other hand, both modifiers led to a similar decrease in the elastic modulus down to close values, 944 and 938 MPa, for PHB films containing 20 wt% PHO and TAC (Figure 5b). Interestingly, at low PHO and TAC content (5 wt%), the strength of PHB blends was improved by around 22 and 27% as against neat PHB. However, when the content of modifiers exceeded 10%, the tensile strength of PHB/PHO films underwent a gradual reduction, while that of PHB/TAC remained in the same range (Figure 5c). Lower strength and elastic modulus values were reported for PHB modified by different plasticizers and toughening agents [12,39]. This may be due to the different preparation conditions and PHO composition, the PHO used in this study being almost a homopolymer, with 95 mol% 3-hydroxyoctanoate units. The eight times increase in the elongation at break is very important given that a small increase in PHB elongation was reported, even for high concentrations of low molecular weight plasticizers [9,10,17]. On the other hand, TAC behaves as a common plasticizer, acting as a spacer between PHB molecules and allowing limited flexibility. The excellent effect of PHO on the ductility of PHB may enable the design of new PHB-based materials with a wider range of mechanical properties, thereby increasing the potential of their application in soft tissue engineering, packaging, or other applications where the balance of ductility and stiffness is a must.

### 3.6. Surface Morphology

**Figure 6:**
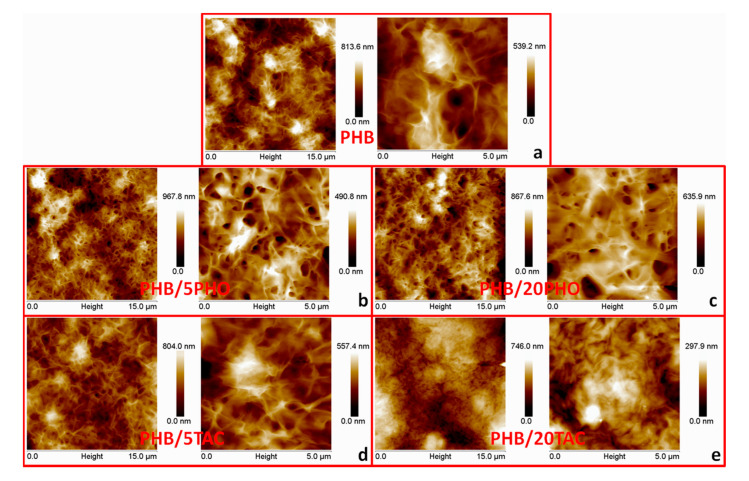
AFM topographic images (height) of neat (**a**) and modified (**b**–**e**) PHB films at 15 (left) and 5 μm (right) scan areas.

The morphology of neat PHB and PHB films containing 5 and 20wt% PHO and TAC, on their surface exposed to air, was investigated by AFM, and representative topographic images at different scan areas are shown in Figure 6a–c. Neat PHB film displayed a well-known spherulitic morphology consisting of an organized fibrous structure corresponding to the lamellar stacks (Figure 6a) [17,37,40]. All the analyzed samples showed a clearly edge-on lamellar growth. The addition of 5wt% PHO or TAC resulted in small changes; however, the crystalline structure was clearly noticed (Figure 6b,d). No significant changes were observed at a high concentration of PHO (PHB/20PHO) compared to neat PHB; however, compared to PHB/5PHO, the crystal structure was less well organized. Indeed, 20 wt% PHO may cause some restrictions in the PHB spherulites’ growth compared to PHB/5PHO due to the limitations in chain mobility. On the contrary, a high concentration of TAC plasticizer led to a disordered structure and a different rearrangement on the surface of PHB (Figure 6e). A high degree of disorder is observed in the crystalline structure of PHB/20TAC (Figure 6d) and the blurred images suggest the migration of TAC to the surface of the film, forming a pellicle. These morphological changes can be better observed in the AFM images taken at a lower scan area of 5μm in Figure 6e. The PHB surface roughness was quantitatively characterized by the root mean square roughness (Rq) on the basis of four unprocessed topographic AFM images of 15 × 15 μm^2^. An Rq value of 160 ± 1.2 nm was found for neat PHB film. After PHO addition, the Rq increased to 213 ± 9.8 nm for PHB/5PHO and to 168 ± 4.9 nm for PHB/20PHO. The increased Rq value found in PHB film with low PHO content may be attributed to the spherulites formed on the surface [41]. Since the rate of nucleation and crystal growth depends on the supersaturation of the employed solvent and obtaining conditions, either nucleation or growth may be dominant over the other, and crystals of different sizes and shapes may be obtained [42]. Contrarily, TAC alters the surface roughness of PHB; lower Rq values were obtained with increasing TAC content, 145 ± 8.5 nm and 124 ± 0.7 nm for PHB/5TAC and PHB/20TAC films. This may be due to the migration of small molecules of TAC to the upper surface of the PHB film, which was further investigated by XPS analysis.

### 3.7. Surface Properties by X-ray Photoelectron Spectroscopy

**Figure 7:**
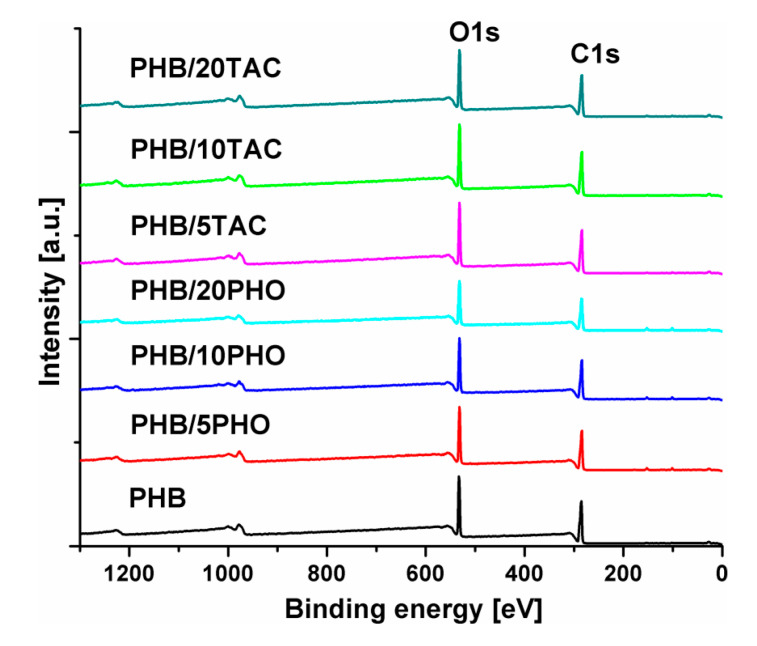
XPS survey spectra of neat and plasticized PHB films.

XPS analyses show the surface modification and composition of PHB after the addition of modifiers. XPS survey spectra of neat PHB and PHB blends are presented in Figure 7. The results show the presence of O and C as main elements and also some impurities consisting of Si and metal traces (less than 2%). Therefore, the lower thermal stability of PHB-PHO blends (Figure 1) may be due to these impurities, which catalyze the depolymerization reaction of PHB by β-elimination [30]. The corresponding elementary content and carbon to oxygen ratios are given in Table 4.

The higher C/O values in comparison to the theoretical ones, measured for all the analyzed PHB films, can be due to hydrocarbon impurities on the surface of the films, as observed in the case of other biopolymers [43]. However, the differences between experimental and theoretical C/O values are lower in the case of modified samples compared to neat PHB, except for PHB/5TAC, where a value close to that of PHB in the limit of experimental error was noticed. Indeed, PHB was used as a powder, which has a high ability to absorb impurities, and the addition of solid or liquid modifiers decreased this tendency. Considering that PHO has double the number of C atoms compared to PHB, the lower difference between experimental and theoretical C/O ratios confirms that PHO did not migrate out of the samples, in good agreement with the TGA and AFM results. Unfortunately, TAC has a C/O ratio close to that of PHB and no conclusion can be drawn from the survey spectra regarding migration. Therefore, high-resolution spectra were analyzed.

**Figure 8:**
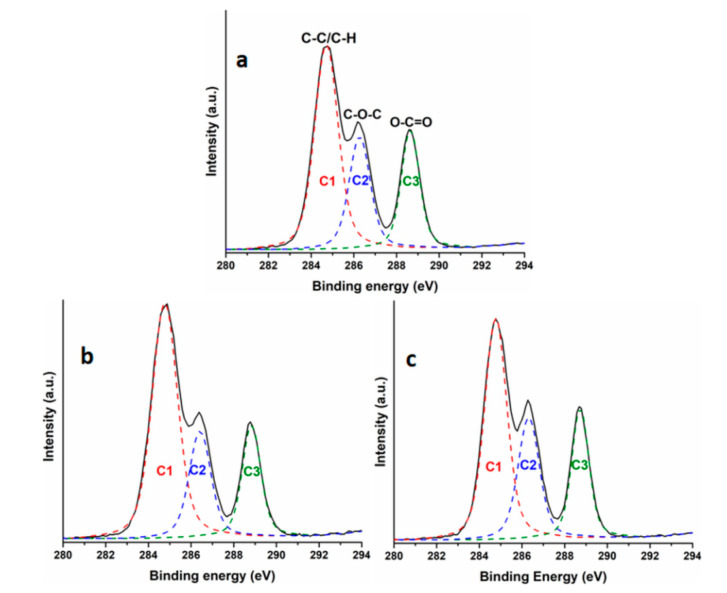
XPS C1s core-level spectrum for neat PHB (**a**), PHB/20PHO (**b**), and PHB/20TAC films (**c**).

Figure 8a–c shows the XPS C1s spectra of neat PHB and PHB films containing the maximum PHO and TAC content.

**Figure 9:**
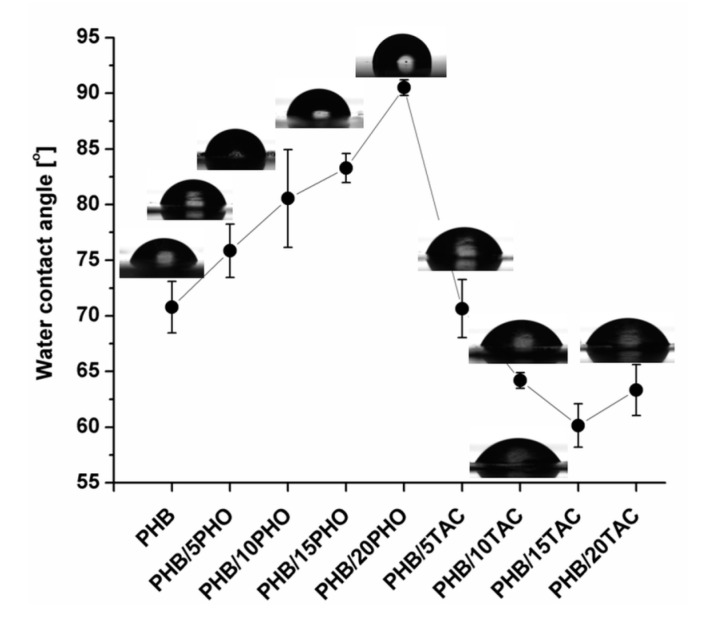
Water contact angle measurements for PHO and TAC modified PHB films.

Three peaks were detected in all PHB films: the peak at 284.7 eV (C1), attributed to carbon in hydrocarbons (C–C, C–H), the peak at 286.3 eV (C2), associated with the ether bond (C–O), and the peak at higher binding energy, 288.6 eV (C3), corresponding to the carbonyl (C=O) bond. The values of binding energies of these carbon species are in good agreement with the ones found in the literature [44,45]. Based on the proportion of these carbon species, the surface concentration of the different chemical bonds was calculated and the results are given in Table 4. As expected, an increase in C1 proportion was observed with the increase in PHO content in PHB/PHO due to the higher number of C-C bonds in the polymeric modifier and no significant variation in PHB/TAC samples (Table 4). However, the increase in C2 and C3 proportion in PHB/TAC samples shows a higher proportion of TAC on the surface of the films; therefore, the migration of TAC occurred even at room temperature. Contact angle measurements (Figure 9) also show increased hydrophilicity on the surfaces of these films, confirming the XPS results.

In summary, the XPS findings support the results of AFM and TGA analysis related to the migration of low molecular weight TAC plasticizer.

### 3.8. In Vitro Biocompatibility

**Figure 10:**
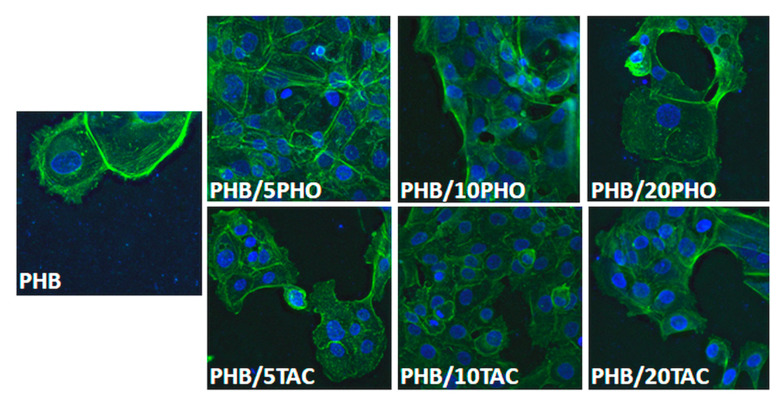
L929 cell adherence and morphology of cells on PHO and TAC modified PHB films.

The attachment and proliferation of L929 cells on the neat and modified PHB films were studied over a period of 1 and 9 days. L929 cells scarcely adhered to the PHB films, regardless of the type and concentration of the toughening agent used, and all tested materials exhibited roughly the same behavior after one day. Images from day 9 (Figure 10) showed a greater number of adhered cells, characterized by fibroblast-like spindle shapes, usually packed in clusters, and tightly anchored on the surfaces. All the tested films allowed the adherence of L929 cells, although some differences were noticed between the modified PHB films. These were related to both the surface roughness determined by AFM (Figure 6) and the surface hydrophobicity characterized by contact angle (Figure 9).

Thus, moderate cell adhesion was observed in the case of plain PHB and PHB-20PHO (Figure 10). The moderate cell adhesion in the case of plain PHB is a well-known behavior [46]. Meanwhile, a high amount of PHO (PHB/20PHO) led to increased hydrophobicity and a contact angle of around 90°, due to the surface-oriented hydrocarbon-containing moieties [47]. This was further reflected in the weaker cell adhesion. Previous studies have shown that better cell adhesion was observed on surfaces with a medium value of the contact angle, of around 70° [46,48]. Therefore, very high or a very low hydrophobicity is not effective in cell adhesion. Indeed, the samples with the best cell adhesion were those with a contact angle between 65° and 75° (PHB/5PHO, PHB/10PHO, PHB/10TAC), therefore around 70°, as generally accepted [46,48]. However, the different behavior of PHB/5TAC supports the idea that there are other factors that influence the biocompatibility. Similarly, PHB/20TAC, with a contact angle close to that of PHB-10TAC, showed slightly poorer cell adhesion. It is worth mentioning that PHB/5TAC and PHB/20TAC showed very low roughness compared to PHB and PHB-PHO films, and it was reported that a lower degree of roughness is not favorable to cell attachment and proliferation [49,50].

The chemistry of the surface has also an influence on cell adhesion [49]. The migration of TAC to the surface, highlighted by AFM, contact angle, and TGA results, may contribute to differences in the response of the surface to cell adhesion and proliferation. In summary, the modified PHB films’propensity to support cell proliferation was demonstrated at day 9, when cells tend to cover greater areas, proving good biocompatibility in vitro.

### 3.9. Pro-Inflammatory Effect Evaluation

**Figure 11:**
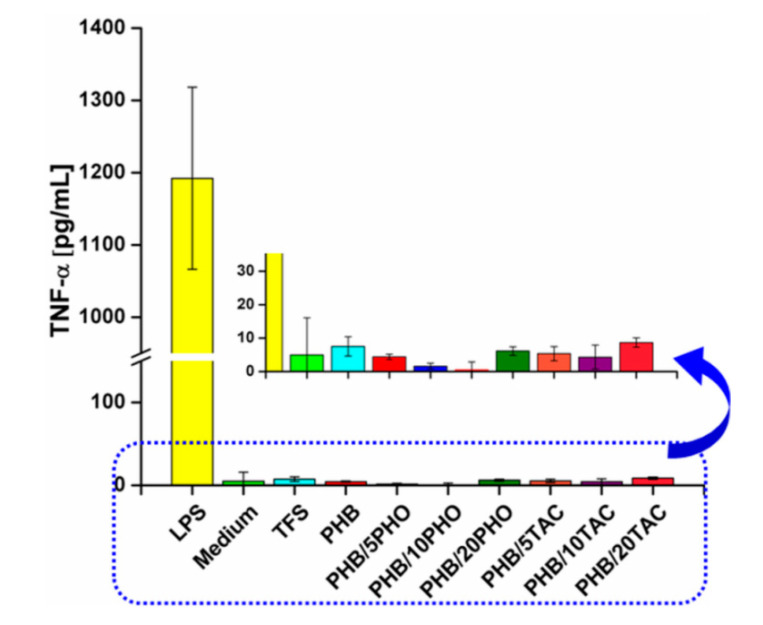
Effect of modified PHB film elution sampleson TNF-α level after an incubation period of 24 h.

To obtain more insight into the biomedical suitability of the modified PHB films, the evaluation of inflammatory susceptibility was assessed by measuring the release of TNF-α (Figure 11).

As can be noticed from Figure 11, TNF-α concentration was much lower compared to the LPS sample and similar to the control or cell culture medium control, regardless of the composition of the PHB films. Moreover, the presence of TNF-α was undetectable in the case of PHB/10PHO. These preliminary results indicate that PHO and TAC modified PHB films are suitable for biomedical applications since very low levels of inflammatory TNF-α cytokine were detected.

## 4. Conclusions

PHB films were prepared by solution-casting using two different bio-based toughening agents, a low-molecular one (TAC) and a polymeric one (PHO). The two modifiers induced different effects on the thermal stability of PHB films and especially on *T*_5%_. A much higher decrease in *T*_5%_, ranging from 39 to 72 °C, was observed in the case of PHB/TAC compared to PHB/PHO films. This different thermal behavior was related to the relatively low boiling point of TAC, which is released from the film starting from 130 °C. Both modifiers led to a slight decrease in the glass transition temperature of PHB along with increased crystallinity and crystal size, favoring the chain motions and the organization of the crystalline phase of PHB. However, PHO led to much better flexibility and elongation of break in PHB/PHO films compared to TAC, being a more efficient toughening agent. In addition, no migration was noticed in the case of PHB/PHO films by AFM and XPS surface analyses. On the contrary, the blurred AFM topographic images of PHB/20TAC suggest the migration of the plasticizer, also confirmed by the increased C2 and C3 proportions in XPS analysis and the decrease in the contact angle value. All the plasticized PHB films supported L929 cell attachment and proliferation, demonstrating good biocompatibility in vitro. The differences in cell adhesion noticed between the plasticized PHB films were related to both surface roughness and surface wettability. In addition, significantly lower levels of TNF-α were detected for PHB films than those of the controls, regardless of the type of the modifier. It can be concluded that PHO is a better modifier for PHB intended for biomedical applications or food packaging due to the lack of migration in the surrounding medium, better thermal stability at the processing temperature of PHB, and better flexibility of the films.

## Figures and Tables

**Table 1:** TGA parameters for neat PHB and plasticized PHB films.

Sample	*T*_5%_ (°C)	*WL*_220_ (%)	*T_d_*_1_ (°C)	*T_d_*_2_ (°C)	*R*_500_ * (%)
PHB	261.3	0.3	-	281.4	0.7
PHB/5PHO	252.2	0.4	-	275.0	0.7
PHB/10PHO	245.6	0.5	-	269.3	0.5
PHB/15PHO	246.6	1.1	-	271.0	0.7
PHB/20PHO	227.5	2.2	-	255.0	1.1
PHO	262.3	0.2	-	287.8	2.8
PHB/5TAC	213.1	5.0	202.5	275.5	0.4
PHB/10TAC	180.8	7.0	191.1	279.5	0.1
PHB/15TAC	174.6	17.0	195.9	277.4	0.3
PHB/20TAC	176.1	17.0	198.5	278.2	0.3

* *R*_500_ is the residue at 500 °C.

**Table 2:** DSC results determined from the heating and cooling cycles for neat and plasticized PHB films.

Sample	Heating			Cooling		
	*T_m_*_1_/*T_m_*_2_ (°C)	Δ*H*_1_/Δ*H*_2_ (J/g)	*X_c_* (%)	*T_g_* (°C)	*T_c_* (°C)	Δ*H_c_* (J/g)
PHB	163.3/173.6	17.8/59.3	53	7.1	88.9	59.0
PHB/5PHO	164.2/173.7	21.1/52.0	53	4.7	82.4	52.4
PHB/10PHO	163.03/173.3	16.7/52.4	53	6.6	84.4	50.6
PHB/20PHO	163.9/173.0	18.9/41.9	52	4.0	75.6	40.9
PHB/5TAC	158.3/172.1	14.5/58.4	53	4.5	83.6	51.6
PHB/10TAC	153.8/169.9	11.9/58.7	54	−1.5	76.5	49.5
PHB/20TAC	150.0/169.2	7.6/61.9	60	−1.5	77.0	51.2

**Table 3:** XRD interplanar distances and crystallite sizes for neat PHB and PHB films with 20 wt% modifier.

Sample	*d*_020_ (nm)	*d*_110_ (nm)	*d*_021_ (nm)	*d*_101_ (nm)	*d*_111_ (nm)	*d*_121_ (nm)	*d*_040_ (nm)	*d*_002_ (nm)	*D*_020_ (nm)	*D*_110_ (nm)	*D*_040_ (nm)
PHB	0.6565	0.5259	0.4426	0.4140	0.3948	0.3497	0.3282	0.2928	14.8	7.5	8.8
PHB/20PHO	0.6580	0.5253	0.4428	0.4123	0.3927	0.3498	0.3279	0.2958	17.4	13.3	14.5
PHB/20TAC	0.6565	0.5240	0.4426	0.4118	0.3922	0.3597	0.3279	0.2923	15.9	13.3	20.4

**Table 4:** Atomic concentrations of C and O elements and relative proportion of C species from C 1s fitting on the surface of neat PHB and plasticized PHB films acquired from XPS scans.

Sample			Atomic Percentage (%)			C1s Peak Fit Atomic Percentage (%)		
	C1s	O1s	C/O Experimental	C/O Theoretical	Difference	C1 [C-C]	C2 [C-O]	C3 [C=O]
**PHB**	69.99	30.01	2.33	2.00	+0.33	51.89	25.16	22.95
**PHB/5PHO**	70.19	29.81	2.35	2.10	+0.25	52.81	25.67	21.52
**PHB/10PHO**	70.90	29.10	2.44	2.20	+0.20	55.46	23.89	20.64
**PHB/20PHO**	70.82	29.18	2.43	2.40	+0.03	57.96	22.10	19.94
**PHB/5TAC**	70.49	29.51	2.39	2.03	+0.36	50.91	26.05	23.03
**PHB/10TAC**	69.89	30.11	2.32	2.05	+0.27	50.22	26.63	23.15
**PHB/20TAC**	70.39	29.61	2.38	2.10	+0.28	50.77	26.37	22.86

## Supplementary Materials

The following are available online at https://www.mdpi.com/2073-4360/12/11/2446/s1, Figure S1: Detailed images of DSC cooling curves of *T_g_* steps for neat PHB and PHB containing PHO (a) and TAC (b); Figure S2: DSC second heating curves for neat PHB and PHB containing PHO (a) and TAC (b).

## References

[1] Nielsen T.D., Hasselbalch J., Holmberg K., Stripple J. (2020). Politics and the plastic crisis: A review throughout the plastic life cycle. WIREs Energy Environ..

[2] Torres-Giner S., Hilliou L., Melendez-Rodriguez B., Figueroa-Lopez K.J., Madalena D., Cabedo L., Vicente A.A., Lagaron J.M. (2018). Melt processability, characterization, and antibacterial activity of compression-molded green composite sheets made of poly(3-hydroxybutyrate-co-3-hydroxyvalerate) reinforced with coconut fibers impregnated with oregano essential oil. Food Packag. Shelf Life.

[3] Bonartsev A.P., Bonartseva G.A., Reshetov I.V., Kirpichniko M.P., Shaitan K.V. (2019). Application of Polyhydroxyalkanoates in Medicine and the Biological Activity of Natural Poly(3-Hydroxybutyrate). Acta Nat..

[4] Mangeon C., Michely L., Rios de Anda A., Thevenieau F., Renard E., Langlois V. (2018). Natural Terpenes Used as Plasticizers for Poly(3-hydroxybutyrate). ACS Sustain. Chem. Eng..

[5] Panaitescu D.M., Nicolae C.A., Gabor A.R., Trusca R. (2020). Thermal and mechanical properties of poly(3-hydroxybutyrate) reinforced with cellulose fibers from wood waste. Ind. Crops Prod..

[6] Panaitescu D.M., Frone A.N., Chiulan I. (2016). Nanostructured biocomposites from aliphatic polyesters and bacterial cellulose. Ind. Crops Prod..

[7] Audic J., Lemiègre L., Corre Y. (2014). Thermal and mechanical properties of a polyhydroxyalkanoate plasticized with biobased epoxidized broccoli oil. J. Appl. Polym. Sci..

[8] Erceg M., Kovacic T., Klaric I. (2005). Thermal degradation of poly(3-hydroxybutyrate) plasticized with acetyl tributyl citrate. Polym. Degrad. Stab..

[9] Seoane I.T., Manfredi L.B., Cyras V.P. (2017). Effect of two different plasticizers on the properties of poly(3-hydroxybutyrate) binary and ternary blends. J. Appl. Polym. Sci..

[10] Wang L., Zhu W., Wang X., Chen X., Chen G.Q., Xu K. (2008). Processability modifications of poly(3-hydroxybutyrate) by plasticizing, blending, and stabilizing. J. Appl. Polym. Sci..

[11] Requena R., Jiménez A., Vargas M., Chiralt A. (2016). Effect of plasticizers on thermal and physical properties of compression-moulded poly[(3-hydroxybutyrate)-co-(3-hydroxyvalerate)] films. Polym. Test..

[12] Garcia-Garcia D., Fenollar O., Fombuena V., Lopez-Martinez J., Balart R. (2017). Improvement of mechanical ductile properties of poly(3-hydroxybutyrate) by using vegetable oil derivatives. Macromol. Mater. Eng..

[13] Aliotta L., Vannozzi A., Panariello L., Gigante V., Coltelli M.-B., Lazzeri A. (2020). Sustainable Micro and Nano Additives for Controlling the Migration of a Biobased Plasticizer from PLA-Based Flexible Films. Polymers.

[14] Arrieta M.P., López J., López D., Kenny J.M., Peponi L. (2015). Development of flexible materials based on plasticized electrospun PLA–PHB blends: Structural, thermal, mechanical and disintegration properties. Eur. Polym. J..

[15] Kurusu R.S., Siliki C.A., David É., Demarquette N.R., Gauthier C., Chenal J.M. (2015). Incorporation of plasticizers in sugarcane-based poly(3-hydroxybutyrate)(PHB): Changes in microstructure and properties through ageing and annealing. Ind. Crops Prod..

[16] Maiza M., Benaniba M.T., Quintard G., Massardier-Nageotte V. (2015). Biobased additive plasticizing Polylactic acid (PLA). Polímeros.

[17] Panaitescu D.M., Nicolae C.A., Frone A.N., Chiulan I., Stanescu P.O., Draghici C., Iorga M., Mihailescu M. (2017). Plasticized poly(3-hydroxybutyrate) with improved melt processing and balanced properties. J. Appl. Polym. Sci..

[18] Arrieta M.P., López J., López D., Kenny J.M., Peponi L. (2016). Biodegradable electrospunbionanocomposite fibers based on plasticized PLA–PHB blends reinforced with cellulose nanocrystals. Ind. Crops Prod..

[19] Kang H., Li Y., Gong M., Guo Y., Guo Z., Fang Q., Li X. (2018). An environmentally sustainable plasticizer toughened polylactide. RSC Adv..

[20] Corrêa M.C.S., Branciforti M.C., Pollet E., Agnelli J.A.M., Nascente P.A.P., Avérous L. (2012). Elaboration and Characterization of Nano-Biocomposites Based on Plasticized Poly(Hydroxybutyrate-Co-Hydroxyvalerate) with Organo-Modified Montmorillonite. J. Polym. Environ..

[21] Dias A.M.A., Marceneiro S., Braga M.E.M., Coelho J.F.J., Ferreira A.G.M., Simões P.N., Veiga H.I.M., Tomé L.C.I., Marrucho M., Esperança J.M.S.S. (2012). Phosphonium-based ionic liquids as modifiers for biomedical grade poly(vinyl chloride). Acta Biomater..

[22] Nerkar M., Ramsay J.A., Ramsay B.A., Kontopoulou M. (2014). Melt Compounded Blends of Short and Medium Chain-Length Poly-3-hydroxyalkanoates. J. Polym. Environ..

[23] Panaitescu D.M., Lupescu I., Frone A.N., Chiulan I., Nicolae C.A., Tofan V., Stefaniu A., Somoghi R., Trusca R. (2017). Medium Chain-Length Polyhydroxyalkanoate Copolymer Modified by Bacterial Cellulose for Medical Devices. Biomacromolecules.

[24] Dufresne A., Vincendon M. (2000). Poly(3-hydroxybutyrate) and Poly(3-hydroxyoctanoate) Blends: Morphology and Mechanical Behavior. Macromolecules.

[25] Chiulan I., Panaitescu D.M., Frone A.N., Teodorescu M., Nicolae C.A., Casarica A., Tofan V., Salageanu A. (2016). Biocompatible polyhydroxyalkanoates/bacterial cellulose composites: Preparation, characterization, and in vitro evaluation. J. Biomed. Mater. Res. A.

[26] Bragg W.H., Bragg W.L. (1913). The reflexion of X-rays by crystals. Proc. R. Soc. London Ser. A.

[27] Patterson A.L. (1939). The Scherrer formula for X-ray particle size determination. Phys. Rev..

[28] Weinmann S., Bonten C. (2019). Thermal and rheological properties of modified polyhydroxybutyrate (PHB). Polym. Eng. Sci..

[29] Kim K.J., Doi Y., Abe H. (2006). Effects of residual metal compounds and chain-end structure on thermal degradation of poly(3-hydroxybutyric acid). Polym. Degrad. Stab..

[30] Kim K.J., Doi Y., Abe H. (2008). Effect of metal compounds on thermal degradation behavior of aliphatic poly(hydroxyalkanoic acid)s. Polym. Degrad. Stab..

[31] Anbukarasu P., Sauvageau D., Elias A. (2016). Tuning the properties of polyhydroxybutyrate films using acetic acid via solvent casting. Sci. Rep..

[32] Malmir S., Montero B., Rico M., Barral L., Bouza R. (2017). Morphology, thermal and barrier properties of biodegradable films of poly (3-hydroxybutyrate-co-3-hydroxyvalerate) containing cellulose nanocrystals. Comp. Part A Appl. Sci. Manuf..

[33] Siracusa V., Ingrao C., Karpova S.G., Olkhov A.A., Iordanskii A.L. (2017). Gas transport and characterization of poly(3 hydroxybutyrate) films. Eur. Polym. J..

[34] Prakalathan K., Mohanty S., Nayak S.K. (2014). Reinforcing effect and isothermal crystallization kinetics of poly(3-hydroxybutyrate) nanocomposites blended with organically modified montmorillonite. Polym. Compos..

[35] Baltieri R.C., Innocentini Mei L.H., Bartoli J. (2003). Study of the influence of plasticizers on the thermal and mechanical properties of poly(3-hydroxybutyrate) compounds. Macromol. Symp..

[36] Iulianelli G.C.V., David G.D.S., dos Santos T.N., Sebastião P.J.O., Tavares M.I.B. (2018). Influence of TiO_2_ nanoparticle on the thermal, morphological and molecular characteristics of PHB matrix. Polym. Test..

[37] Chen J., Wu D., Tam K.C., Pan K., Zheng Z. (2017). Effect of surface modification of cellulose nanocrystal on nonisothermal crystallization of poly(β-hydroxybutyrate) composites. Carbohydr. Polym..

[38] Hong S.G., Gau T.K., Huang S.C. (2011). Enhancement of thecrystallization and thermal stability of polyhydroxybutyratebypolymericadditives. J. Therm. Anal. Calorim..

[39] Garcia-Garcia D., Ferri J.M., Montanes N., Lopez-Martinez J., Balart R. (2016). Plasticization effects of epoxidized vegetable oils on mechanical properties of poly(3-hydroxybutyrate). Polym. Int..

[40] Lin K.W., Lan C.H., Sun Y.M. (2016). Poly[(R)3-hydroxybutyrate] (PHB)/poly(l-lactic acid) (PLLA) blends with poly(PHB/PLLA urethane) as a compatibilizer. Polym. Degrad. Stab..

[41] Lee C.W., Song B.K., Jegal J., Kimura Y. (2013). Cell adhesion and surface chemistry of biodegradable aliphatic polyesters: Discovery of particularly low cell adhesion behavior on poly(3-[RS]-hydroxybutyrate). Macromol. Res..

[42] Sofińska K., Barbasz J., Witko T., Dryzek J., Haraźna K., Witko M., Kryściak-Czerwenka J., Guzik M. (2019). Structural, topographical, and mechanical characteristics of purified polyhydroxyoctanoate polymer. J. Appl. Polym. Sci..

[43] Panaitescu D.M., Frone A.N., Chiulan I., Casarica A., Nicolae C.A., Ghiurea M., Trusca R., Damian C.M. (2016). Structural and morphological characterization of bacterial cellulose nano-reinforcements prepared by mechanical route. Mater. Des..

[44] Wang C., Sauvageau D., Elias A. (2016). Immobilization of Active Bacteriophages on Polyhydroxyalkanoate Surfaces. ACS Appl. Mater. Interfaces.

[45] da Silva M.G., Vargas H., Poley L.H., Rodriguez R.S., Baptista G.B. (2005). Structural impact of hydroxyvalerate in polyhydroxyalkanoates (PHAscl) dense film monitored by XPS and photothermal methods. J. Braz. Chem. Soc..

[46] Lee C.W., Horiike M., Masutani K., Kimura Y. (2015). Characteristic cell adhesion behaviors on various derivatives of poly(3-hydroxybutyrate) (PHB) and a block copolymer of poly(3-[RS]-hydroxybutyrate) and poly(oxyethylene). Polym. Degrad. Stab..

[47] Qu X.H., Wu Q., Liang J., Zou B., Chen G.Q. (2006). Effect of 3-hydroxyhexanoate content in poly(3-hydroxybutyrate-co-3-hydroxyhexanoate) on in vitro growth and differentiation of smooth muscle cells. Biomaterials.

[48] Tamada Y., Ikada Y. (1994). Fibroblast growth on polymer surfaces and biosynthesis of collagen. J. Biomed. Mater. Res..

[49] Boyan B.D., Hummert T.W., Dean D.D., Schwartz Z. (1996). Role of material surfaces in regulating bone and cartilage cell response. Biomaterials.

[50] Surmenev R.A., Chernozem R.V., Syromotina D.S., Oehr C., Baumbach T., Krause B., Boyandin A.N., Dvoinina L.M., Volova T.G., Surmeneva M.A. (2019). Low-temperature argon and ammonia plasma treatment of poly-3-hydroxybutyrate films: Surface topography and chemistry changes affect fibroblast cells in vitro. Eur. Polym. J..

