# Supplementary material for: Low Molecular Weight and Polymeric Modifiers as Toughening Agents in Poly(3-Hydroxybutyrate) Films
Source: Polymers (Basel). 2020 Oct 22;12(11):2446. doi: 10.3390/polym12112446 (PMC7716241; doi:10.3390/polym12112446)
Supplement: Supplementary file 1 [file polymers-12-02446-s001.zip › polymers-972627-supplementary.docx]

Low molecular weight and polymeric modifiers as toughening agents in poly(3-hydroxybutyrate) films

Adriana Nicoleta Frone^1,^*, Cristian Andi Nicolae^1^, Mihaela Carmen Eremia^2^, Vlad Tofan^3^, Marius Ghiurea^1^, Ioana Chiulan^1^, Elena Radu^1^, Celina Maria Damian^4^ and Denis Mihaela Panaitescu^1,^*

^1^ Polymer Department, National Institute for R&D in Chemistry and Petrochemistry ICECHIM, 202 Splaiul Independentei, Bucharest, 060021, Romania; [ciucu_adriana@yahoo.com](mailto:ciucu_adriana@yahoo.com); [ca_nicolae@yahoo.com](mailto:ca_nicolae@yahoo.com); [ghiurea@gmail.com](mailto:ghiurea@gmail.com); [ioana.chiulan@icechim.ro](mailto:ioana.chiulan@icechim.ro); [nina.radu58@yahoo.ro](mailto:nina.radu58@yahoo.ro); [panaitescu@icechim.ro](mailto:panaitescu@icechim.ro);

^2^ National Institute for Chemical Pharmaceutical Research and Development ICCF, 112 Calea Vitan, Bucharest, 031299, Romania; [mihaelaceremia@yahoo.com](mailto:mihaelaceremia@yahoo.com)

^3^ Cantacuzino National Institute of R&D for Microbiology and Immunology, 103 Splaiul Independentei, Bucharest, 050096, Romania; [tofan.vlad@gmail.com](mailto:tofan.vlad@gmail.com)

^4^ Advanced Polymer Materials Group, Faculty of Applied Chemistry and Materials Science, University Politehnica of Bucharest, 1-7 Gheorghe Polizu, Bucharest, 011061, Romania; [celina.damian@yahoo.com](mailto:celina.damian@yahoo.com)

***** Correspondence: [ciucu_adriana@yahoo.com](mailto:ciucu_adriana@yahoo.com); [panaitescu@icechim.ro](mailto:panaitescu@icechim.ro);


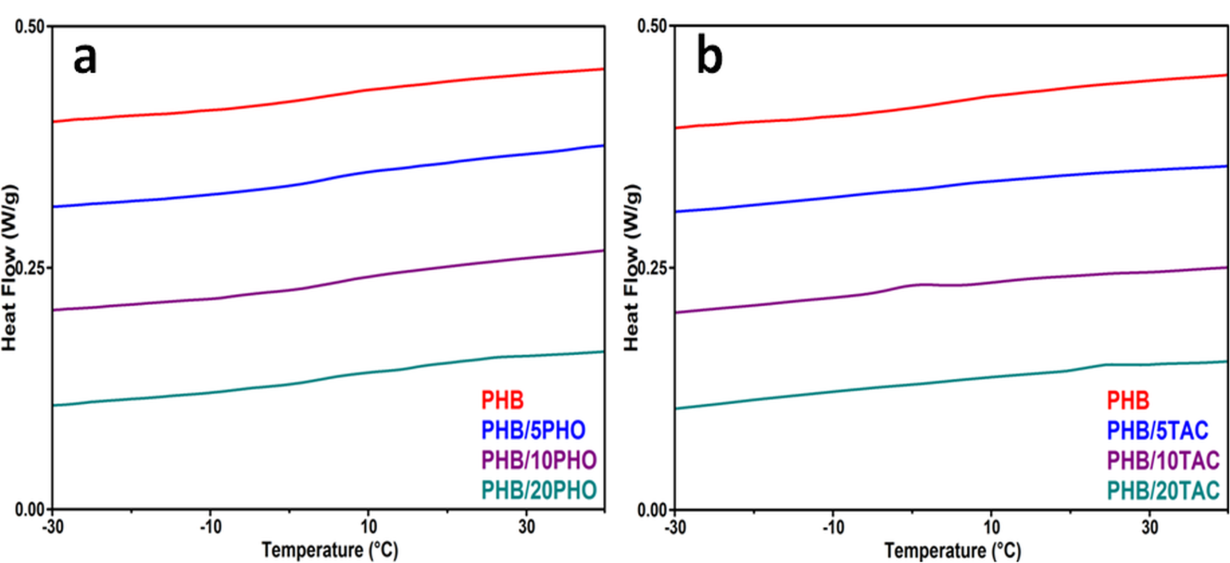


**Figure S1**. Detailed images of DSC cooling curves of *T_g_* steps for neat PHB and PHB containing PHO (a) and TAC (b)


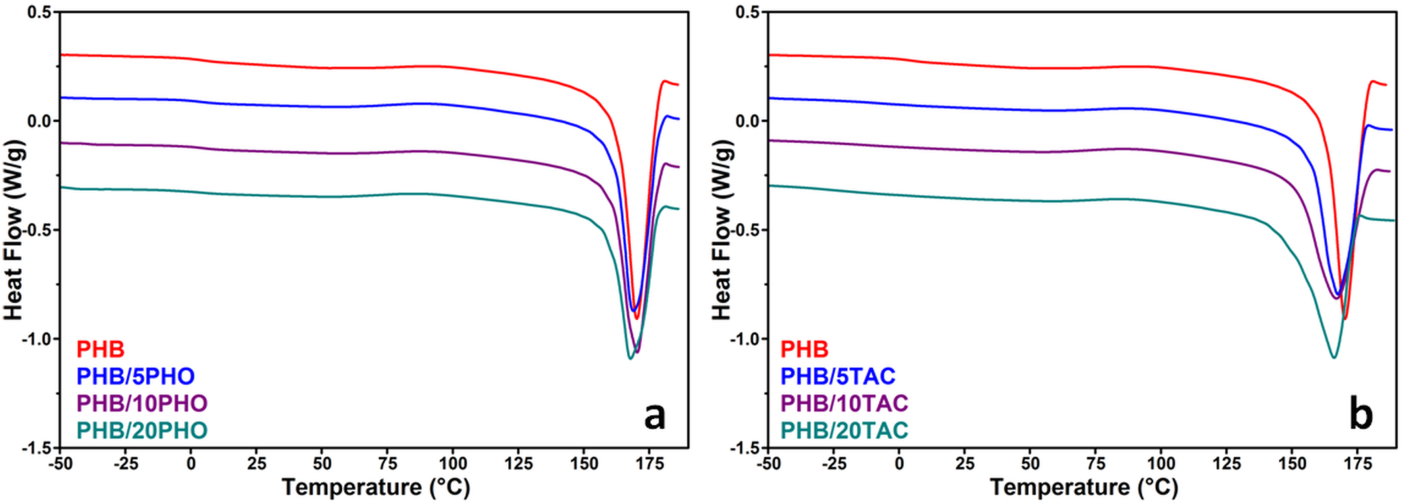
**Figure S2.** DSC second heating curves for neat PHB and PHB containing PHO (a) and TAC (b)
